# Case report: A rare case of coexistence of low-grade appendiceal mucinous neoplasia and goblet cell adenocarcinoma in the appendix

**DOI:** 10.3389/fonc.2024.1313548

**Published:** 2024-03-04

**Authors:** Ping Zhou, Xuejiao Yu, Du He

**Affiliations:** Department of Pathology, West China Hospital, Sichuan University, Chengdu, China

**Keywords:** low-grade appendiceal mucinous neoplasia, goblet cell adenocarcinoma, appendix, coexistence, histopathology

## Abstract

**Background:**

Primary appendiceal tumors are rare. Low-grade appendiceal mucinous neoplasia (LAMN) and goblet cell adenocarcinoma (GCA) account for 20% and 14% of primary appendiceal tumors, respectively. The coexistence of LAMN and GCA is an extremely rare event. This report presents a case of an elderly male patient with an appendiceal tumor composed of LAMN and GCA in the same appendix.

**Case presentation:**

A 72-year-old male patient was admitted to our institution presenting with a history of abdominal pain localized to the right lower quadrant for two months. Abdominal computed tomography (CT) showed a large dilated thickened cystic mass in the appendix, along with a small duodenal diverticulum. Laboratory tests indicated elevated levels of serum carcinoembryonic antigen (CEA) and cancer antigen 199 (CA19-9) markers. The patient underwent a laparoscopic right hemicolectomy and exploration of the duodenal diverticulum, and there was no finding of perforation of the duodenal diverticulum. Focal positivity for chromogranin A (CgA) and synaptophysin (Syn) was observed in the tumor cells of GCA. The final pathological diagnosis revealed the coexistence of LAMN staged pT4a and grade 1 GCA staged pT3 in the appendix. Unfortunately, the patient died due to severe septic shock and circulatory failure secondary to a perforated duodenal diverticulum.

**Conclusions:**

The coexistence of LAMN and GCA are extremely rare in the appendix and may result from the proliferation of two independent cellular lines. The coexistence of distinct neoplasms poses diagnostic and management challenges. Multidisciplinary team discussion may be essential in the effective management of these patients.

## Introduction

The incidence of epithelial appendiceal neoplasms is rare (1), accounting for approximately 0.5% of all gastrointestinal tract tumors (2). However, the incidence has increased over the years (2, 3). The clinical presentation is nonspecific. Many appendiceal tumors are generally asymptomatic and incidentally discovered by appendectomy for appendicitis or an abdominal mass associated with abdominal pain (4). Appendiceal neoplasms were identified in 0.78% of acute appendicitis patients (5). Symptoms of acute appendicitis commonly occur in elderly patients, particularly those with elevated levels of serum tumor biomarkers, thus an appendiceal tumor should be suspected. The preoperative diagnosis of appendiceal neoplasms may be challenging. Ultrasound scans and/or computed tomography (CT) are effective in detecting appendiceal tumors. Ultrasound and CT scans revealing enhanced masses and irregularly thickened walls can be indicators of malignancy (6).

Appendiceal mucinous neoplasms are the second most common tumors after neuroendocrine neoplasms (3). LAMN was identified in 0.6% of all appendectomies, accounted for approximately 20% of appendiceal neoplasms, and represented up to 73% of mucinous epithelial neoplasms (7). LAMN is characterized by mucinous epithelial proliferation with extracellular mucin and pushing invasion and can tend to develop pseudomyxoma peritonei (PMP) (8, 9). Appendectomy was sufficient for LAMN patients without perforation or PMP (2). Cytoreductive surgery (CRS) and hyperthermic intraperitoneal chemoperfusion (HIPEC) are recommended for PMP (9).

Goblet cell adenocarcinoma (GCA), formerly termed goblet cell carcinoid, accounts for 14% of primary neoplasms of the appendix (10) and has demonstrated an incidence of 0.05-0.3 per 100,000 per year among North American registry studies (11). GCA was renamed a separate tumor entity in the 5th World Health Organization (WHO) Classification of Digestive System Tumors (12, 13). GCA is an amphicrine tumor composed of goblet-like mucinous cells, as well as variable numbers of endocrine cells and Paneth-like cells, typically arranged as tubules resembling intestinal crypts (11). There are three grades for GCA, depending on the proportion of low-grade to high-grade tumor components (11): grade 1: >75% tubular or clustered growth, grade 2: 50-75% tubular or clustered growth, and grade 3: <50% tubular or clustered growth. GCA can metastasize via the lymphatic vessels, and right hemicolectomy is a recommended treatment option (2). GCA with regional lymph node or distant metastases generally requires systemic chemotherapy.

However, two or more histologically distinct tumors within the same appendix were an extremely rare that may be considered a coincidental occurrence (7, 14, 15) caused by independent progenitor cells. The coexistence of LAMN and GCA is quite rare (16–20). In this report, we present a case of an elderly male patient with a histologically confirmed diagnosis of coexisting LAMN and GCA in the resected appendix. Furthermore, a review of the coexistence of LAMN and GCA reported in the literature was conducted.

## Case presentation

**Figure 1:**
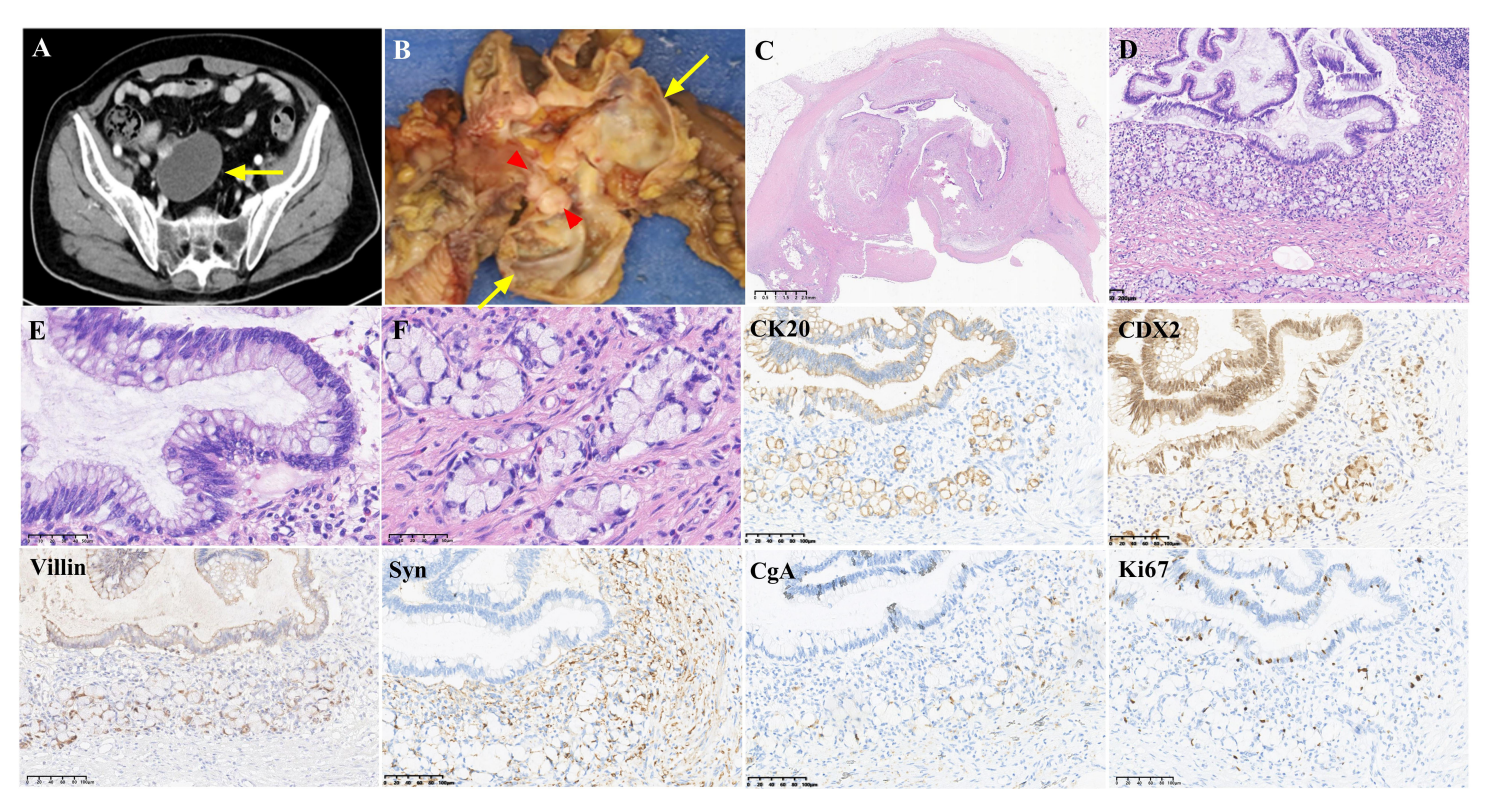
Radiological imaging, histopathological features and immunohistochemical staining of coexistence of low-grade appendiceal mucinous neoplasia (LAMN) and goblet cell adenocarcinoma (GCA) in the appendix. **(A)**. Abdominal CT revealed a large cystic mass in the appendix (arrow), indicating a cystic appendiceal tumor. **(B)**. Gross examination demonstrated a dilated cystic mass (arrows) with accumulation of mucin in the appendix and a nodule (red triangles) in the cystic wall. **(C)**. Low-power field showed a dilated appendix with intramural epithelium pushing invasion in the thickened wall. In addition, lymphoid tissue was markedly absent. (H&E, magnification x10). **(D)**. Histopathological diagnosis of the lesion was consistent with LAMN and GCA. The tumor cells of LAMN and GCA were adjacent. (H&E, magnification x100). **(E)**. LAMN showed low-grade, slightly elongated nuclei and abundant mucin-filled cytoplasm. (H&E, magnification x400). **(F)**. The grade 1 GCA consisted of clusters of cuboidal cells and goblet-like mucinous cells in discrete, clustered units embedded in dense collagen. (H&E, magnification x400). Immunohistochemical staining revealed positive expression of CK20, CDX2 and Villin in the tumor cells of both LAMN and GCA. Focal positivity for Synaptophysin (Syn) and chromogranin (CgA) was observed in GCA tumor cells, while LAMN tumor cells showed negative staining for these markers. The Ki67 index was approximately 5% and 3% in the tumor cells of LAMN and GCA, respectively. (Magnification x200).

A 72-year-old male patient presented to the local hospital with a history of abdominal pain localized to the right lower quadrant for two months. The patient did not present with fevers or chills. Ultrasound and abdominal CT scan showed a dilated appendix with a thickened wall and peri-appendiceal inflammation, indicating an appendiceal mucinous neoplasm or carcinoma. Subsequently, the patient was admitted to our hospital where a new abdominal CT was ordered, revealing a large dilated thickened cystic mass in the appendix measuring 5.9 cm x 4.9 cm (**Figure 1A**). The cystic wall was irregular thickened, and there were nodules in the cystic wall, indicating an appendiceal mucinous neoplasm or adenocarcinoma. Additionally, the abdominal CT scan indicated a duodenal diverticulum with inflammatory infiltration. Physical examination revealed tenderness. Laboratory tests indicated elevated levels of serum carcinoembryonic antigen (CEA) and cancer antigen 199 (CA19-9) markers, 9.12 ng/ml and 111.00 U/ml, respectively, and a normal level of cancer antigen 125 (CA125). Given our strong suspicion of an appendiceal mucinous neoplasm or carcinoma infiltrating the appendiceal wall preoperatively, the patient underwent a laparoscopic right hemicolectomy. The duodenal diverticulum was explored during the surgical procedures, and there was no finding of perforation.

On gross examination (**Figure 1B**), the cystic mass measured 60mm × 50mm was located in the appendix, and the appendix wall was thickened by approximately 1mm to 12mm. The appendix demonstrated dilation as a result of abnormal accumulation of mucin, without evidence of perforation or rupture. A nodule measuring 12mm x 10mm was identified in the cystic wall. All appendiceal tumors were subjected to histologic examination.

Microscopic examination at low-power magnification showed that a dilated appendix with a thickened wall and obvious intraluminal mucin, without evidence of appendix rupture. The lymphoid tissue of the appendix was reduced or absent (**Figure 1C**). No calcification of the wall was observed. There were two distinct and adjacent histological components (**Figure 1D**). The mucinous epithelial proliferated cells replaced the normal epithelial mucosa, and tumors exhibited columnar cytoplasmic mucin vacuoles, which compressed the nucleus (**Figure 1E**). Nuclear atypia is mild, and mitosis is absent. Mucin pools extended to the serosa, and the appendiceal serosa was involved. The lesion was diagnosed as LAMN staged as pT4a due to acellular mucinous deposits. In addition, the adjacent tumor cells grew as nests of goblet cells, and tubules invaded the subserosa of the appendix. Microscopic examination at high magnification showed that the tumors were goblet-like mucinous cells with tumor cell clusters (**Figure 1F**). Tumur cells are distended by large mucin vacuoles that eccentrically displace the nuclei. The tumor consists of more than 75% tubular and clustered growth. The present tumor cells were diagnosed as grade 1 GCA staged pT3. Perineural invasion was present in GCA. Lymphovascular invasion was not observed. Resection margins and lymph nodes were negative. The ileal and colon of the specimen did not show tumor involvement. Immunohistochemical staining results are shown in **Figure 1**. Diffuse staining for cytokeratin 20 (CK20), CDX2 and Villin was positive in the tumor cells of LAMN and GCA. Focal positivity for chromogranin A (CgA) and synaptophysin (Syn) was observed in the tumor cells of GCA. The Ki67 index was approximately 5% and 3% in the tumor cells of LAMN and GCA, respectively. Final pathology revealed the coexistence of LAMN staged pT4a and grade 1 GCA staged pT3 in the appendix.

The patient received anti-infection and nutritional support in the intensive care unit (ICU) following laparoscopic right hemicolectomy. However, three days postoperatively, the patient presented with an acute onset abdominal pain in the right upper quadrant, and drainage of greenish-yellow pus. A new abdominal CT revealed a suspected perforation of the descending duodenal diverticulum. Subsequently, the patient underwent an urgent exploratory laparotomy, revealing a 10mm x 5mm perforated duodenal diverticulum that was promptly repaired. Gram-negative bacteria (carbapenem-resistant *Klebsiella pneumoniae*, CRKP) and fungi were detected in both the abdominal ascites and blood cultures. Despite aggressive treatment, the patient’s clinical condition progressively deteriorated due to severe septic shock and circulatory failure. The patient died on the ninth second postoperative day.

## Discussion

Appendiceal tumors are rare entities. The coexistence of distinct appendiceal tumors is extremely rare and may result from two or more distinct tumor types (7, 16, 20–23). Appendiceal collision tumors are frequently incidentally detected following appendectomy or other conditions, because there are no specific clinical or radiological characteristics. The coexistence of appendiceal tumors was finally diagnosed by pathological examination. The management of collision tumors is complex due to the different behaviors exhibited by distinct histological components. The biological behavior and clinical treatment may be determined by the more aggressive histological components.

The coexistence of LAMN and GCA is quite rare (16, 20, 23). Among a total of 671 cases of carcinoid tumors in the appendix, only 1.9% (13/671) were identified as dual carcinoid/epithelial neoplasms (24), with a goblet cell type in three cases and a mucinous cystadenoma in four cases, however, this article did not provide detailed information of the coexistence of distinct appendiceal tumors. Only 11 cases of GCA and LAMN have been reported in the published English-language literature with available detailed descriptions (16–20). The present report discusses a rare patient with the coexistence of LAMN and GCA. A summary of the similar rare cases is shown in **Table 1**. Eight cases were diagnosed with LAMN and GCA (16, 18–20). There have been extremely rare reported cases of triple synchronous tumors of the appendix: carcinoid, GCA and LAMN in one case (17), and triple synchronous tumors of GCA, LAMN and mucinous carcinoma (MCA) in two cases (19). The female-to-male ratio was 7:4, with a maximal tumor size of 65mm (16). The clinical presentation is not specific. Four cases presented with symptoms resembling acute appendicitis (16, 18, 20), and a pelvic cystic lesion was incidentally detected in one case (17). PMP indicates mucinous neoplasms with spread beyond the appendix. There were two cases with pools of extravasated mucin and tumor cells in the appendiceal walls and serosal surfaces (16), one case with PMP (19) and one case with pseudomucinous tumor formation around the appendix (20).

**Table 1:** The summary of rare cases of coexistence of low-grade appendiceal mucinous neoplasia (LAMN) and goblet cell adenocarcinoma (GCA) reported in the published English-language literature.

Authors, years	Age/Sex	Tumor size (cm)	Symptoms	Surgical treatment	Diagnosis	Peritoneal dissemination	Outcome
**R K Al-Talib. et al (** 16)**, 1995**	54/F	60x15mm	Two months after an attack of appendicitis.	Appendectomy	Combined goblet cell carcinoid and mucinous cystadenoma	Serosal surfaces contained pools of extravasated mucin and tumor cells	NA
	64/F	65x12mm	Four months history of a dull ache in the right iliac fossa which had become increasingly severe over the last week.	Appendectomy	Combined goblet cell carcinoid and mucinous cystadenoma	Serosal surfaces contained pools of extravasated mucin and tumor cells	NA
**Khaled O Alsaad. et al (** 18)**, 2009**	46/F	Appendiceal wall measured 1.5cm maximally	Severe acute pain in the right iliac fossa and periumbilical region	Right hemicolectomy	Combined goblet cell carcinoid and mucinous cystadenoma	None	NA
**DENISE NG et al (** 19)**, 2014**	58/F	NA	NA	Cytoreductive surgery	GCA+LAMN	PMP	NA
	46/M	NA	NA	Appendectomy	GCA+LAMN	None	NA
	65/F	NA	NA	Appendectomy	GCA+LAMN+MCA	None	NA
	65/M	NA	NA	Appendectomy	GCA+LAMN+MCA	None	NA
	56/M	NA	NA	Appendectomy	GCA+LAMN	None	NA
	59/F	NA	NA	Appendectomy	GCA+LAMN	None	NA
**Fabio Carboni et al (** 17)**, 2020**	54/F	30×24×23mm	A pelvic cystic lesion incidentally detected on ultrasonography	Laparoscopic right hemicolectomy with bilateral oophorectomy	Carcinoid, goblet cell carcinoma and LAMN	None	NED, 9 months
**Yang, Ruiting et al (** 20)**, 2023**	72/M	NA	Eriumbilical pain and discomfort and intermittent attack of symptoms for more than 8 months	Appendectomy	GCA+LAMN	Pseudomucinous tumor formation around the appendix	NED, 80 days
**The current case, 2022**	72/M	60x50mm	Abdominal pain localized to the right lower quad-rant for two months	Laparoscopic right hemicolectomy	GCA+LAMN	None	Dead, 12 days after the first surgery

M, male; F, female; LAMN, low-grade appendiceal mucinous neoplasia; GCA, goblet cell adenocarcinoma; MCA, mucinous carcinoma; NA, not available; NED, no evidence of death; PMP, pseudomyxoma peritonei.

Surgical treatment appears to be a safe and feasible approach for appendiceal tumors. All 11 patients underwent surgical treatment (16–20), and the two available reported patients had a favorable prognosis following successful surgical treatment (17, 20). One patient presented with GCA invading the serosa and LAMN with mucus invading the muscular wall and pseudomucinous tumor formation around the appendix, with no evidence of death for 80 days (20). The other patient presented with triple synchronous tumors of the appendix: carcinoid invading into the mesoappendix, GCA invading the muscular layer and LAMN without signs of infiltration of the appendiceal wall, with no evidence of death for 9 months (17). Eight patients underwent appendectomy, two patients underwent right hemicolectomy, and one patient with PMP underwent surgical cytoreduction. The extent of surgery depends on the tumor location, size, and histological type. The role of appendectomy or right hemicolectomy in management the coexistence of appendiceal cancers remains controversial. Appendectomy is considered sufficient when there are no risk factors for Tis (LAMN) and T3 disease, while right hemicolectomy may be sufficient if there are no risk factors for T4a LAMN (25). The Chicago Consensus Working Group recommends right hemicolectomy for GCA regardless of T stage (26). GCA can metastasize via the lymphatic vessels and the bloodstream and should be treated by oncological right hemicolectomy (2). Appendectomy alone appears adequate for stage I disease, and right hemicolectomy is appropriate for T4 tumors or stage II to III disease provided that it can be performed with minimal risk (27). Kowalsky et al. (28) reported that a survival benefit with right hemicolectomy was identified for pT3-T4 tumors on appendectomy but not for pT1-T2 tumors. Furthermore, lymph node positivity rates were 1.1%, 2.1%, 9.9%, and 29.1% for T1-T4, respectively (28). Tsang et al. (29) demonstrated that lymph node positivity rates were 0%, 15%, and 34% for pT2-T4 on right hemicolectomy, respectively. These findings suggest that right hemicolectomy should be considered as the standard surgical treatment for appendiceal GCA staged pT3-T4 (30). The present report describes an elderly male patient with an appendiceal tumor diagnosed as LAMN staged pT4a and grade-1 GCA staged pT3 in the appendix. The patient underwent a laparoscopic right hemicolectomy. LAMN pT4a due to acellular mucinous deposits had a 3% risk of developing peritoneal recurrence (9). Unfortunately, postoperative morbidity was present in this case. The patient died due to severe septic shock and circulatory failure secondary to a perforated duodenal diverticulum. Infection in the present patient was associated with CRKP infection and fungal infection. Severe septic shock remains the leading cause of mortality in critically ill patients. Gram-negative bacteria, gram-positive bacteria, and fungi were isolated in 65%, 25%, and 10% of the 269 severe sepsis patients in the surgical intensive care units, respectively. The most prevalent species were *Klebsiella pneumoniae* (31). CRKP infection is a life-threatening disease with high rates of morbidity and mortality. Older age and septic shock were risk factors for death after CRKP infection (32). The coexistence of distinct neoplasms poses diagnostic and management challenges. Multidisciplinary team discussion may be essential in the effective management of these patients.

Appendiceal goblet cells and mucinous neoplasms are biologically unique tumors (33). However, a previous study suggested that appendiceal goblet cell carcinoid and mucinous neoplasms are closely associated tumors, may share a common tumor stem cell with the potential for multiple lineage differentiation and are associated with alterations in WNT signaling (19). The present report describes an elderly male patient with an appendiceal tumor who presented with LAMN staged pT4a and grade-1 GCA staged pT3 in the appendix by right hemicolectomy. The tumor consists of two adjacent and distinct components, lacking any transitional zone, which may be considered as a coincidental occurrence resulting from the proliferation of two independent cellular lines. Histopathological diagnosis of the distinct components within the same neoplasm is important for the management of coexistent appendiceal tumors. Further investigation is needed to determine the significance of this rare combination.

## Conclusion

The coexistence of distinct neoplasms within the same appendix is extremely rare and poses diagnostic and management challenges. The definitive diagnosis is established by histopathological examination and immunohistochemical staining after surgery. This report discusses a rare case who presented with two synchronous appendiceal tumors of LAMN and GCA, suggesting the proliferation of two independent cellular lineages.

## Data availability statement

The original contributions presented in the study are included in the article/supplementary material. Further inquiries can be directed to the corresponding author.

## Ethics statement

Written informed consent was obtained from the individual(s) for the publication of any potentially identifiable images or data included in this article.

## Author contributions

PZ: Writing – original draft, Writing – review & editing. XY: Methodology, Software, Writing – original draft. DH: Project administration, Validation, Writing – review & editing.
